# mHealth Interventions for Self-Harm: Scoping Review

**DOI:** 10.2196/25140

**Published:** 2021-04-30

**Authors:** Bethany Cliffe, Jessica Tingley, Isobel Greenhalgh, Paul Stallard

**Affiliations:** 1 Department for Health University of Bath Bath United Kingdom; 2 Child and Adolescent Mental Health Services Oxford Health NHS Foundation Trust Bristol United Kingdom

**Keywords:** mHealth, self-harm, digital interventions, self-injury, NSSI, mobile phone

## Abstract

**Background:**

Self-harm is a growing issue with increasing prevalence rates; however, individuals who self-harm do not often receive treatment. Mobile health (mHealth) interventions are a possible solution to some of the barriers that individuals face when seeking support, and they have also been found to be effective in improving mental health. Thus far, reviews of mHealth interventions for self-harm have been limited by study type. Therefore, we determined that a broader scoping review will provide a more exhaustive understanding of mHealth interventions for self-harm.

**Objective:**

This scoping review aims to identify mHealth interventions for self-harm within the literature, understand the types and features of interventions that have been developed and evaluated, highlight research findings around mHealth interventions for self-harm, and determine what outcomes are typically used to assess the efficacy of interventions.

**Methods:**

A search was conducted using Embase, PubMed, PsycINFO, PsycEXTRA, Web of Science, and the Cochrane Library. Studies were included if they described an mHealth intervention designed to have a direct (ie, if the intervention was designed for self-harm or for people who self-harm) or indirect (ie, if self-harm was measured as an outcome) treatment effect and if the paper was available in English. There were no exclusion criteria based on the study design.

**Results:**

A total of 36 papers were included in the review, and most of them were randomized controlled trials published within the last 4 years. The interventions were mostly smartphone apps and calling or texting services, with 62% (21/34) having underlying therapeutic models to inform the intervention content. They were generally shown to be promising and appealing, but only 5 were widely available for use. Outcomes focused on a reduction of self-harm and suicidality, mood, and the users’ experiences of the intervention. Samples were typically nondiverse, and there was limited variety in the study designs and in the measurements of self-harm recovery.

**Conclusions:**

Promising and appealing mHealth interventions have been developed but are not widely available. Research could benefit from greater diversity as well as a broader and more nuanced understanding of recovery from self-harm.

## Introduction

### Self-harm

The National Institute for Health and Care Excellence defines self-harm as any act of self-injury or poisoning, irrespective of the motivation behind the act [1]. It is a growing concern that can have great physical, psychological [2], and societal [3] costs. Notably, self-harm has been identified as a significant and persistent predictor of suicide [4]. In recent years, the lifetime prevalence of self-harm in the general English population has seen a sharp increase from 2.4% in 2000 to 6.4% in 2014; this was most common in young adult females who reported an increase in prevalence from 6.5% to 19.7% [5]. Furthermore, given that individuals are often reluctant to disclose their self-harm behaviors due to the shame and stigma associated with it [6], rates of self-harm may be even higher than what these figures suggest [7].

### Help for Self-harm

Tørmoen et al [8] surveyed 11,440 young people aged 14-17 years in Norway and found that only 34% of those who had self-harmed had ever sought professional help, indicating that help-seeking is low among those who self-harm [9,10]. Concerns over being perceived as “attention-seeking” or “crazy” and difficulty talking about their self-harm behaviors have been identified as some of the barriers to seek support by adolescents [11]. Furthermore, a lack of effective interventions for self-harm creates barriers to receive support when an individual seeks professional help. The National Institute for Health and Care Excellence guidelines advise against the use of pharmacological treatments for self-harm, instead recommending psychological interventions tailored to self-harm that may involve problem solving, cognitive behavioral or psychodynamic elements [12]. Despite this, to date, there is limited high-quality evidence suggesting that psychological or pharmacological interventions for self-harm are effective [13,14]. Moreover, increased pressure on services and the resulting difficulties with the availability and accessibility of these interventions can further prevent individuals from receiving support [15].

### Use of Mobile Health

The use of mobile health (mHealth) may help overcome the barriers to treatment accessibility and availability. mHealth is a branch of eHealth, defined by the Global Observatory for eHealth as “medical and public health practice supported by mobile devices, such as mobile phones, patient monitoring devices, personal digital assistants (PDAs), and other wireless devices” [16]. Given the ubiquity of mobile phone ownership [17,18], providing mental health support in this way has the potential to reach many individuals who may not be receiving help for self-harm. mHealth offers multiple possibilities, including self-help smartphone apps, SMS text messaging with a support service, physical symptom tracking through wearable technologies, and receiving virtual therapy [19]. Clough and Casey [20] found that mHealth users felt that receiving virtual mHealth therapy was more beneficial compared with face-to-face therapy, particularly highlighting the freedom they felt to be completely open and honest with their therapist. mHealth tools also have merit as standalone interventions, with some studies reporting reductions in symptoms of mental health difficulties, including anxiety [21], schizophrenia [22], depression [23], and borderline personality disorder [24].

Studies investigating the efficacy of mHealth interventions for managing self-harm have also been reviewed but they are limited. Witt et al [25] identified only one study that included outcome measures of an mHealth intervention for self-harm, whereas Melia et al [26] identified 2. These reviews focused on randomized controlled trials (RCTs) and pre- and poststudies. Arshad et al [27] focused more closely on self-harm and identified 22 studies; however, this was still limited by their decision to exclude qualitative studies and those where self-harm was not the primary outcome. A broader scoping review will help to identify more mHealth tools available for managing self-harm and broaden our knowledge of them.

### Aims

This scoping review aims to (1) identify mHealth interventions for self-harm within the literature, (2) understand the types and features of interventions that have been developed and evaluated, (3) highlight research findings around mHealth interventions for self-harm, and (4) determine what outcomes are typically used to assess the efficacy of interventions.

## Methods

### Overview

A detailed methodology can be found in the review protocol [28]. The following databases were searched in April 2020: Embase, PubMed, PsycINFO, PsycEXTRA, Web of Science, and the Cochrane Library. The reference lists of all papers identified in the searches were also screened. Multimedia Appendix 1 describes the full and detailed search strategy.

After duplicates were removed, the titles and abstracts were initially screened according to the aims of this review and were progressed for a full screening if they met the following inclusion criteria: (1) the study described an mHealth intervention (eg, SMS text messaging, phone calls, or websites accessible through a mobile device) designed to have a direct (ie, if the intervention was designed for self-harm or for people who self-harm) or indirect (if self-harm was measured as an outcome) treatment effect and (2) the paper was in English.

The full texts of the papers that met these criteria were then screened. Before screening the title and abstract, a pilot screening was performed by all 3 reviewers (BC, JT, and IG) on 20 papers selected at random. An interrated reliability check of at least 75% agreement was required to progress the papers to full screening. Initially, 80% agreement was achieved, and the remaining papers were briefly discussed until 100% agreement was achieved. Each paper was screened by at least two reviewers, with a third reviewer resolving any inconsistencies. During the title and abstract screening, there was 98% agreement between the 2 reviewers on each paper, with the others being discussed and resolved again. The full texts of the progressed papers were screened for eligibility.

### Data Charting Process

The reviewers extracted predefined data regarding the study details (eg, year and country), participants (eg, number, age, and ethnicity), type of mHealth intervention, study design, measures, and outcomes. Both before and after data extraction, consistency was checked between reviewers using a random sample of papers. Data extraction was an iterative process in which categories were added or amended in accordance with the aims of the review.

## Results

### Identify mHealth Interventions for Self-Harm Within the Literature

**Figure 1:**
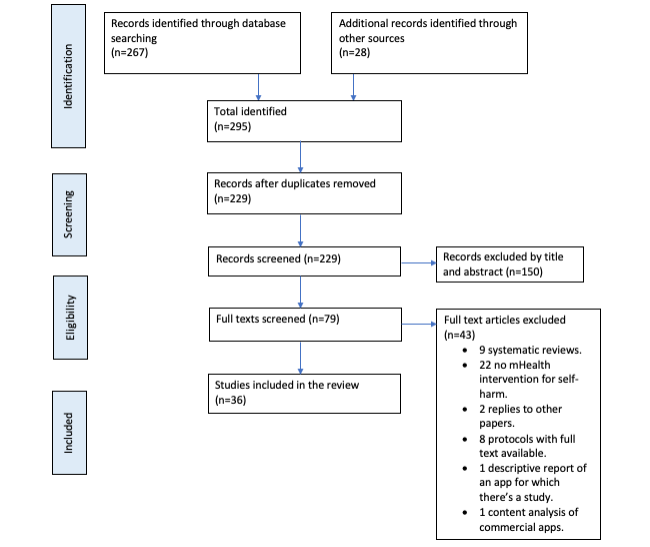
Flowchart of search results. mHealth: mobile health.

The search results are summarized in Figure 1. A total of 295 papers were identified. After duplicates were removed, 78% (229/295) of titles and abstracts were left to be screened. About 35% (79/229) of titles and abstracts progressed to a full-text screening, resulting in 54% (43/79) of papers being excluded. Of these, 51% (22/43) did not present an mHealth intervention for self-harm, 21% (9/43) were systematic reviews that did not identify any papers not already identified by our search, and 19% (8/43) were protocols for studies for which a full text had since become available. Of the remaining 4 papers, 2 (5%) were replies or comments on other studies, 1 (2%) was a content analysis of apps that were commercially available with no references to an evidence base, and 1 (2%) was a description of an app that had already been identified. A total of 36 papers met the inclusion criteria and are summarized in Table 1.

**Table 1:** Summary of papers (N=36).

Intervention details	Type of intervention	Design	Sample	Type of self-harm	Self-harm measure used	Development	Availability	Improvement in self-harm
Rebound (2019) [29]: *Accessible anytime*^a^	Social network with support from peer workers and clinical psychologists	RCT^b^ (protocol)	Youth (aged 14-27 years) in recovery from major depressive disorder–Australia	Suicidal and nonsuicidal self-harm	Risk taking and self-harm inventory	Collaboration with consumers, youth representatives, and clinicians	Not widely available	No data
SIAM (2014) [30]: *9 texts sent, staggered for 6 months following A&E^c^ discharge*	Supportive and monitoring texts	RCT (protocol)	Adults discharged after suicide attempt–France	Suicidal self-harm	Columbia-Suicide Severity Rating Scale	Developed by the authors	Not widely available	No data
A Virtual Hope Box (2015; 2017) [31,32]: *Accessible anytime*	Smartphone app to help with coping, distraction, relaxation, and positive thinking	Proof of concept; RCT	Army veterans at risk of self-harm or suicide–United States	Suicidal self-harm; suicidal and nonsuicidal self-harm	Semistructured interview; Beck Scale for Suicidal Ideation and Columbia-Suicide Severity Rating Scale	Developed by the Department of Defense National Centre for Telehealth and Technology, with feedback from target users	Free to download	Yes; no
EpxDepression (2017) [33]: *Daily EMA^d^ for 2-4 months*	Text-based EMA that notifies care team of risk	Proof of concept	Adolescents–United States	Suicidal self-harm	Last question of PHQ^e^-9	Clinicians, researchers, and biostatisticians with feedback from target end users	Available for health care providers on subscription	Yes
Imaginator (2020) [34]: *Accessible anytime*	Smartphone app to promote self-management	RCT	Youth (aged 16-25 years) currently self-harming–United Kingdom	Suicidal and nonsuicidal self-harm	Strength of motivation for reducing self-harm scale, craving experience questionnaire for self-harm, self-harm imagery interview. Self-harm, frequency, severity, and self-efficacy for control were measured with items developed by the enhancer	Codeveloped with youth with lived experience of self-harm	Not widely available	Yes
Therapeutic Evaluative Conditioning (2016) [35]: *Accessible anytime*	Smartphone app to decondition self-harm	RCT	Adults with self-harm–international	Suicidal and nonsuicidal self-harm	Self-injurious thoughts and behaviors interview	Developed by the authors	No longer available	Yes, but did not persist at follow-up
BlueIce (2018) [36,37]: *Accessible anytime*	Smartphone app to help manage urges to self-harm	Pre-post phase 1 trial	Adolescents currently self-harming or at risk of self-harming attending CAMHS^f—^United Kingdom	Nonsuicidal self-harm	Semistructured interview; Self Harm data from clinical records	Coproduced with youth with lived experience of self-harm, with input from clinical staff, academics, and app developers	Available for CAMHS on subscription	Yes
Unnamed (2018) [38]: *Accessible anytime*	Smartphone app for mood monitoring and distraction	Development study	Aged 18-25 years—Australia	Nonsuicidal self-harm	N/A^g^	Designed with target users and clinicians	Not widely available	No data
ClinTouch CareLoop enhance management (2014) [39]: *Accessible anytime; 44 daily ecological momentary assessment alerts*	Smartphone app for mood monitoring and alerting care team of risk	RCT (protocol)	Adults with psychotic disorders—United Kingdom	Suicidal and nonsuicidal self-harm	Unclear	Codeveloped with service users, clinicians, academics, and health professionals	Free to download	No data
Unnamed (2016) [40]: *52 text messages sent twice weekly*	Supportive and informative texts	Intervention study	Adults with suicidal ideation—Japan	Nonsuicidal self-harm	Researchers developed their own questionnaire measuring the presence of self-harm	Psychiatry specialists	Not widely available	Yes
A-CHESS (2017) [41]: *Accessible anytime*	Smartphone app containing safety plan, social network, resources, and interaction dashboard with their therapist	Qualitative study of experience	Presented to emergency department following self-harm—Canada	Suicidal and nonsuicidal self-harm	Last question of PHQ-9, hospital presentations for self-harm	Developed by the network for improvement of addiction treatment with user feedback from focus groups	Available for health care providers on subscription	Yes
Safe Storage Intervention (2015) [42]: *Texts sent daily*	Social contact via phone conversations and texts	Cost-effective analysis (protocol)	Participants recruited to another study–Sri Lanka	Suicidal and nonsuicidal self-harm	Hospital presentations and coroner’s data	No data	Not widely available	No data
Brief mobile treatment [43]: *10 staggered phone calls after discharge for 24 weeks; audio messages accessible anytime; text reminders sent weekly*	Phone calls to monitor mood, meditation audio messages, and text reminders of treatment elements	RCT	Aged 15-74 years admitted to hospital after self-harm—Sri Lanka	Suicidal self-harm	Beck Scale for Suicidal Ideation	No data	Not widely available	No
No name (2013) [44]: *Staggered texts that start daily and decline gradually to weekly over 3 months*	Supportive text messages	RCT	Adults in A&E after self-harm—Ireland	Suicidal and nonsuicidal self-harm	Unclear—self-harm repetition	No data	Not widely available	No data
No name (2017) [45]: *Accessible anytime*	Smartphone app to provide care messages, resources, health care contacts, and self-help exercises	RCT (protocol)	Adult in A&E after self-harm—Hong Kong	Suicidal and nonsuicidal self-harm	Hospital presentations and coroner’s data	No data	Not widely available	No data
Unnamed (2018) [46]: *12-month treatment period, no further detail*	Smartphone app to augment delivery of problem-solving therapy	RCT (protocol)	Men in A&E after self-harm–Canada	Suicidal and nonsuicidal self-harm	Hospital presentations	No data	Not widely available	No data
TeenTEXT (2016) [47]: *Receiver sets schedule of when texts will be received*	Supportive texts written by the receiver	Feasibility study	Adolescents currently self-harming—United Kingdom	Nonsuicidal self-harm	N/A	Designed with service users with history of self-harm, carers, and clinicians	Not widely available	No data (terminated)
DBT^h^ Coach (2016) [48]: *Accessible anytime*	Smartphone app that provides coaching in DBT skills	Pilot	Individuals seeking DBT—United States	Suicidal and nonsuicidal self-harm	SITBI^i^, semistructured interviews	Involved DBT experts, target end users, and their clinicians	Available for users on subscription	Yes
Unnamed (2019) [49]: *Delivered over 10-12 sessions*	Audio or video calls to deliver problem-solving CBT^j^	RCT	Aged 16-30 years, depression and self-harm—location unknown	Nonsuicidal self-harm	Urgency Perseverance Premeditation Sensation-seeking Impulsive Inventory urgency subscale	No data	Not widely available	No data (terminated)
MyPlan (2017) [50]: *Accessible anytime*	Smartphone app to store safety plan	RCT (protocol)	Experiencing self-harm—Denmark	Suicidal and nonsuicidal self-harm	Self-reported, no further information	Developed by Skovgaard Larsen et al [51]	Yes (in Denmark and Norway)	No data
SMS SOS (2019) [52]: *Staggered texts that start 48 h after discharge from A&E, declining to monthly for 12 months*	Supportive text messages	RCT (protocol)	In A&E after self-harm—Australia	Suicidal and nonsuicidal self-harm	Hospital presentations	Developed with people with lived experience of self-harm or mental health problems	Not widely available	No data
Crisis Text Line (2020) [53]: *Accessible anytime*	Texting with a crisis counselor	Trend analysis	No criteria—international	Suicidal and nonsuicidal self-harm	N/A	Crisis text line is a global organization	Available globally	N/A
SPARX (2020) [54]: *Must complete program within 6 weeks*	Game-style smartphone app	RCT (protocol)	Year 8 school students—Australia	Suicidal and nonsuicidal self-harm	Self-harm questionnaire	Developed with young people	Yes (in New Zealand)	No data
ERITA (2018) [55]: *12 weeks to complete 11 modules*	Website and companion app to receive treatment for emotion regulation	Pilot	Adolescents with self-harm—Sweden	Nonsuicidal self-harm	Deliberate self-harm inventory	Developed by the authors	Not widely available	Yes
CATCH-IT^k^ (2009) [56]: *Recipients had 3 phone calls, no further information*	Therapy provided via website with motivational interviewing phone calls	RCT	Aged 14-21 years with subthreshold depression—United States	Nonsuicidal self-harm	PHQ-A	Developed by the authors	Not widely available	Yes
Unnamed (1999) [57]: *Accessible anytime for 6 months following discharge*	Telephone crisis consultation with on-call psychiatrist	RCT	Adult inpatients with self-harm—United Kingdom	Nonsuicidal self-harm	Hospital presentations	No data	Not widely available	No
Unnamed (2020) [58]: *A module per week for 6 weeks*	Web-based intervention to provide CBT-based modules	RCT	Turkish adults with suicidal ideation—United Kingdom and Netherlands	Suicidal self-harm	Self-harm questionnaire	Developed by van Spijker et al [59] adapted by the authors	Not yet, but will be if findings are positive	Yes
Unnamed (2018) [60]: *Encouraged to write 5* *min* *a day for 28 days*	Web-based diary	RCT	Adults on an online forum who self-harm—international	Suicidal and nonsuicidal self-harm	SITBI	Developed by the author	Technique can be adopted	Yes
Uncut (2014) [61]: *Ecological momentary assessment*	App to track mood and practice DBT skills, therapist can monitor progress	Development study	Self-harm experts—Austria, Germany, and United States	Nonsuicidal self-harm	N/A	Developed with international psychological experts	No data	No data
Living under control (2017) [62]: *A module per week for 6 weeks*	Web-based intervention providing 6 modules around managing thoughts and feelings	RCT (protocol)	Adults—Denmark	Suicidal and nonsuicidal self-harm	Hospital records and self-report questionnaire (no further information)	Developed by van Spijker et al [63] with mental health professionals who work with people experiencing suicidality	Not yet, but will be if findings are positive	No data
Crisis Care (2017) [64]: *Accessible anytime*	Smartphone app containing coping skills, distraction activities, and a help me now section	Pilot study	Adolescent psychiatry outpatients—United States	Suicidal self-harm	N/A	Developed by the author	Not widely available	Yes
Monsenso (2020) [65]: *Accessible anytime*	Smartphone app providing DBT skills and mood monitoring; links with clinicians database	RCT (protocol)	Adults with borderline personality disorder and self-harm—Denmark	Suicidal and nonsuicidal self-harm	Self-harm inventory	Developed by the author	Available for health care providers on subscription	No data
Unnamed (2019) [66]: *No details yet*	Supportive text messages	Development study	Adolescents with self-harm—China	Nonsuicidal self-harm	N/A	Developed with youth with lived experience of self-harm	No data	No data
Unnamed (2020) [67]: *Unlimited access every evening*	Peer-supported hotline	Trend analysis	Teenagers—United States	Suicidal and nonsuicidal self-harm	N/A	Founded by mental health professionals	Available to teenagers in the United States	N/A

^a^Italics refer to the intervention duration.

^b^RCT: randomized controlled trial.

^c^A&E: accident & emergency.

^d^EMA: ecological momentary assessment.

^e^PHQ: patient health questionnaire.

^f^CAMHS: Child and adolescent mental health services.

^g^N/A: not applicable.

^h^DBT: dialectical behavior therapy.

^i^SITBI: self-injurious thoughts and behaviors interview.

^j^CBT: cognitive behavioral therapy.

^k^CATCH-IT: Competent Adulthood Transition with Cognitive Behavioural Humanistic and Interpersonal Training.

The 36 papers related to 35 separate studies, 2 of which were published from the same study with one paper detailing the quantitative findings [36] and the other the qualitative findings [37]. They were therefore not removed as duplicates; however, as the study details were the same, the study characteristics were merged to not present the same information twice.

A total of 12 papers reported protocols. The corresponding authors were contacted to request any update or preliminary findings; a preprint paper was received from one, so the protocol was replaced with this and the data were extracted. The remaining 11 protocols had no further data at the time of this review (April-June 2020).

The most common study design was the RCT (20/35, 57%), with most papers published between 2014 and 2020 (33/35, 94%). Studies were most commonly conducted in Europe (14/35, 40%) and North America (7/35, 20%). Most studies measured self-harm using various self-report questionnaires (18/35, 51%) and looked at both suicidal and nonsuicidal self-harm (19/35, 54%).

#### Participants

The participant data are summarized in Table 2. Sample sizes ranged from 3 to 122,909, involving mostly clinical cohorts (24/35, 69%), and approximately half were adult samples (18/35, 51%), with one focusing specifically on young adults (aged 18-25 years). None of the studies included children aged <12 years and adults aged >65 years. Gender was reported in 18 papers, with the majority including more females than males (14/18, 78%); only 1 study included a nonbinary participant (1/18, 6%). Most papers provided no data on ethnicity (25/35, 71%) and those that did recruited mostly White people (8/10, 80%). Presenting problems screened for or required as inclusion criteria among the samples were primarily self-harm (26/35, 74%), depression (22/35, 63%), suicidal ideation (15/35, 43%), and suicide attempts (15/35, 43%).

**Table 2:** Participant characteristics (n=35).^a^

Characteristic		n (%)	Study
**Age (years)**			
	Adults	18 (54)	[30-32,35,38-41,44-46,48,57,58,60-62,65]
	Adolescents (up to 18 years)	8 (23)	[33,36,37,47,54,55,64,66,67]
	Adolescents and adults	6 (17)	[29,34,43,49,52,56]
	No age restriction	1 (3)	[50]
	Unknown	2 (5)	[42,53]
**Population**			
	Clinical	24 (69)	[29,30,32,33,36-41,43-50,52,55-57,64-66]
	Community	9 (26)	[31,35,42,53,54,58,60,62,67]
	Clinical and community	1 (3)	[34]
	Mental health professionals	1 (3)	[61]
**Presenting problems or inclusion criteria**			
	Self-harm	26 (74)	[29,30,34-37,40,41,43-50,52,53,55-58,60,62,65-67]
	Depression	22 (63)	[29,31-34,36-38,40,41,43,45,48-50,53-56,60,62,66,67]
	Anxiety	13 (37)	[29,31,32,34,36,37,41,48,49,53-55,66,67]
	Suicidal ideation	15(43)	[29,30,32,34,35,40,43-45,50,53,58,60,62,64]
	Suicide attempt	15 (43)	[29,30,32,34,35,44-46,48,50,53,57,60,64,65]
	Borderline personality disorder	5 (14)	[31,32,48,55,65]
	Psychosis	4 (11)	[32,39,40,66]
	Substance or alcohol use	4 (11)	[32,41,43,57]
	Eating disorders	2 (6)	[31,55]
	Sleep disorders	2 (6)	[32,54]
	Neurological disorders	1 (3)	[32]
	Somatoform disorder	1 (3)	[32]

^a^Grist et al (2018) [36] and Stallard et al (2018) [37] are separate papers from the same study.

### Understand the Types and Features of Interventions That Have Been Developed and Evaluated

#### Characteristics of mHealth Interventions

Intervention characteristics are summarized in Table 3 and relate to 34 interventions. As mentioned earlier, another 2 papers reported on different trials of the same intervention. Most studies described apps (15/34, 44%) or texting or calling services (13/34, 38%), and most interventions required a mobile phone (16/34, 47%) or a smartphone (11/34, 32%), whereas the rest required any internet-enabled device (4/34, 12%) or an iPod touch (1/34, 3%). Approximately half of the interventions did not include any face-to-face support (16/34, 47%); of these, 9 were standalone interventions (9/16, 56%), 7 included an element of human support provided digitally (ie, texts from a clinician; 7/16, 21%), and 2 exclusively provided digital support (ie, a hotline; 2/16, 13%). A range of underpinning therapeutic models informing the intervention content were reported, with 21 studies (21/34, 62%) citing at least one approach and cognitive behavioral therapy being the most common (10/34, 29%). Supportive messages or phone calls were the most common elements among the interventions (14/34, 41%). Only 3 (9%) papers specified that the interventions contained a safety plan.

**Table 3:** Intervention characteristics (n=34).^a^

Intervention characteristics		n (%)	Study
**Intervention type**			
	Apps	15 (44)	[31,32,34,36,38,39,41,45,48,50,54,61,64,65]
	Texting or calling services	13 (38)	[30,33,40,42-44,47,49,52,53,57,66,67]
	Websites or web-based therapies	4 (12)	[55,56,58,62]
	Web-based diary	1 (3)	[60]
	Social network	1 (3)	[29]
**Device**			
	Mobile phone	16 (47)	[30,33,39,40,42-45,47,49,52,53,57,65-67]
	Smartphone	11 (32)	[31,32,34-38,41,46,50,54,55,60,61,64]
	Any internet-enabled device	4 (12)	[29,56,58,62]
	iPod touch (or iPhone)	1 (3)	[48]
**Human support included**			
	Face-to-face provided	12 (35)	[31,32,34,36-38,41,43,46-48,50,52,65]
	Digital support provided	7 (21)	[29,49,53,55,57,58,67]
	No support	9 (27)	[30,33,35,44,54,60,62,64,66]
	Both compared	2 (6)	[45,56]
	Not specified	4 (12)	[39,40,42,61]
**Underpinning therapeutic model**			
	Cognitive behavioral therapy	10 (29)	[31,32,36-38,47,49,54,56,58,62,64]
	Dialectical behavior therapy	6 (18)	[31,32,36-38,48,61,65]
	Cognitive	3 (9)	[29,34,50]
	Behavioral	3 (9)	[35-37,56]
	Mindfulness	2 (6)	[29,36,37]
	Problem-solving therapy	2 (6)	[41,46]
	Acceptance and commitment therapy	1 (3)	[56]
	Interpersonal psychotherapy	1 (3)	[56]
	Autobiographic self enhancement	1 (3)	[60]
	Not specified	13 (38)	[30,33,39,40,42-45,52,53,57,66,67]
**Features**			
	Supportive messages or phone calls	14 (41)	[30,33,34,40,42-45,47,52,53,57,66,67]
	Coping skills	9 (27)	[40,48,54,56,58,61,62,64,65]
	Mood diaries	8 (24)	[34,36-39,45,60,61,65]
	Links to helplines, services, or caregivers	8 (24)	[33,40,41,44-46,53,64]
	Problem-solving techniques	6 (18)	[29,41,43,46,54,56]
	Alerts to clinicians	6 (18)	[33,39,41,47,61,65]
	Mood lifting or physical activities	6 (18)	[34,36,37,44,54,56,64]
	Information and psychoeducation	5 (15)	[29,41,44,54,65]
	Relaxation and meditation	5 (15)	[31,32,36,38,45,64]
	Mindfulness	5 (15)	[29,31,32,36,37,48,58]
	Thought challenging	5 (15)	[36,37,54,56,58,62]
	Photos, music, and other media	4 (12)	[31,32,34,36,37,41]
	Medication and intervention reminders	4 (12)	[34,39,40,43]
	Social and peer support	3 (9)	[29,41,67]
	Safety plan	3 (9)	[41,50,62]
	Distraction methods	3 (9)	[31,32,38,64]
	Games	3 (9)	[31,35,54]

^a^Bush et al (2015) [31] and Bush et al (2017) [32] relate to the same intervention; Grist et al (2018) [36] and Stallard et al (2018) [37] are separate papers from the same study.

#### Development and Availability

Half of the mHealth interventions were developed with multiple collaborators (17/34, 50%), including mental health professionals (10/34, 29%), target end users (11/34, 32%), and companies or organizations (3/34, 9%). Most interventions were not currently available to the public (20/34, 59%). Conversely, only 15% (5/34) are publicly available, whereas others are available for purchase by health care professionals or services (4/34, 12%) or users (1/34, 3%). Table 1 provides further details on the intervention development and availability.

### Highlight Research Findings Around mHealth Interventions for Self-Harm

#### Study Findings

Of the 19 papers that reported outcomes, 14 (74%) reported positive findings postintervention. Of the 5 studies that did not report positive findings, 2 (26%) were terminated during recruitment due to a lack of feasibility. One study noted that this was due to high levels of depression and the reluctance of participants who self-harmed to engage with mental health services [49], whereas the other suggested it was a good intervention but Child and Adolescent Mental Health Services was the wrong setting due to clinicians’ time constraints [47]. Furthermore, 3 studies reported no significant effect of the intervention on self-harm recovery [32,43,57], one of which cited past episodes of self-harm as a barrier to efficacy [57].

Clinician and parental attitudes were typically favorable toward the interventions [31,32,38,41,47,64], with only 2 papers reporting concerns [38,47]. The identified benefits include promoting self-efficacy [32,58], helping difficult disclosure [33], immediate access [43], time and cost benefits [55], encouraging help-seeking [40], being useful in crises [64,66], and having a positive influence on the therapeutic alliance in the face-to-face element within blended approaches [41,58]. Barriers were not commonly mentioned in the papers, although 2 papers did note that digital interventions that were administered by a mental health worker posed challenges due to the lack of engagement with mental health services among people who self-harm [47,49].

### Determine What Outcomes Are Typically Used to Assess the Efficacy of Interventions

The study outcomes are summarized in Table 4. Most studies had multiple outcomes related to self-harm (21/35, 60%), suicide attempts (19/35, 54%), suicidal ideation (16/35, 46%), intervention experience (16/35, 46%), and engagement with the intervention (13/35, 37%). Other mental health issues such as depression (16/35, 46%) and anxiety (9/35, 26%) were also considered. Outcomes related to self-harm mostly focused on episode frequency (14/21, 67%), whereas others focused on repeated presentations to hospital (5/21, 24%) or self-harm thoughts or urges (4/21, 19%).

**Table 4:** Study outcomes (n=35).^a^

Outcome		n (%)	Study
**Mental health**			
	SH^b^ frequency	14 (40)	[29,34-37,39,40,42,44,45,54-60,62]
	Presentations to hospital for SH	5 (14)	[40,45,46,52,57]
	SH thoughts or urges	4 (11)	[34,48,56,60]
	SH (specifics unclear)	2 (6)	[50,58]
	Suicidal ideation	16 (46)	[29,30,32,34,35,40,43-45,49,50,54,58,60,62,65]
	Suicide attempts	19 (54)	[29,30,32,34,35,40,42,43,45,46,48-50,52,57,58,60,62,65]
	Depression	16 (46)	[29,30,34,36-38,43,45,49,50,53,54,56,58,60,62,66]
	Anxiety	9 (26)	[29,30,34,36,37,49,53,54,58,62]
	Eating disorders	3 (9)	[30,53,54]
	Borderline personality disorder	2 (6)	[55,65]
	Psychosis	1 (3)	[54]
**Other well-being**			
	Other mental well-being	7 (20)	[29,48,53-55,60,62]
	Interpersonal issues	7 (20)	[29,32,43,45,49,53,54]
	Hopelessness	6 (17)	[44,45,49,50,58,62]
	Sleep	3 (9)	[29,33,54]
	Alcohol or substance use	3 (9)	[43,53,54]
	Quality of life	3 (9)	[29,58,62]
	Other self-destructive behaviors	2 (6)	[54,55]
**Intervention feasibility and acceptability**			
	Experience	16 (46)	[29-34,36,37,44,47,48,50,54,58,60,62,64,66]
	Engagement	13 (37)	[29,31-34,39,44,45,48,53,54,56,67]
	Health care costs	1 (3)	[46]

^a^Grist et al (2018) [36] and Stallard et al (2018) [37] are separate papers from the same study.

^b^SH: self-harm.

## Discussion

### Principal Findings

This scoping review identified 36 papers relating to 34 separate mHealth tools for managing self-harm. Papers were primarily RCTs and protocols published in Europe or North America within the last 6 years. This recent increase in papers reflects the growing interest in developing evidence-based mHealth interventions to improve access to psychological therapies. The large number of protocols suggests that this trend will continue as more findings are published in the coming years. Participants were mostly White adult females recruited from clinical populations, with only one nonbinary participant across all the studies, and many did not report the ethnicity of their participants. This is concerning given the high prevalence of self-harm found in both ethnic [68] and gender minorities [69]. It is possible that other nonbinary or gender-diverse individuals participated in these studies, but they were not truly represented in the way that the researchers assessed or reported participant demographics.

Depression and anxiety were the 2 most highly studied comorbidities, which is consistent with research suggesting strong correlations between these disorders and self-harm [70]. The interventions most commonly studied were not blended with any face-to-face support and were mostly text or call-based services or apps used on mobile phones or smartphones. Considering the ubiquity of both mobile phones [17,71] and smartphones [71], this is a positive finding and suggests that there are indeed mHealth interventions that could be more widely accessed. Interestingly, no interventions designed for use on other devices, such as wearable technologies, have been identified. This is despite research suggesting that they are acceptable for treating mental health issues among those who do not typically engage with mental health services [72]. However, this is still a relatively new area, and wearable devices designed to treat or help manage mental health difficulties are predicted to increase over the next few years [73].

Several studies did not specify any underpinning therapeutic models informing the content of their intervention, which raises concerns given the unhelpful and even dangerous advice that has been found in other freely available mental health apps [74]. Similarly, less than a third of the interventions were developed with individuals who have lived experience of self-harm, despite evidence suggesting that this could lead to more effective interventions being developed. Given the expertise of those with lived experience of self-harm, their input is essential [75,76].

Most studies testing interventions reported overall promising findings, suggesting that mHealth can be a viable tool for people struggling with self-harm. The study outcomes largely promoted a reduction in self-harm frequency over and above others, such as a reduction in self-harm urges or severity. Although reduced frequency is a common measure of self-harm recovery within research, it has been argued that it is not advisable to rely solely on this; a reduction in self-harm episodes may mean that each episode has become more severe or has been replaced with a different type of self-destructive behavior [77]. Overall, there needs to be a more consistent framework to assess outcomes from self-harm research, with further research looking into the maintenance of positive outcomes. Similarly, there was considerable variation in the tools used to measure self-harm across the studies. This makes comparisons between studies difficult; therefore, greater consistency may also be beneficial.

Another commonly observed finding was the clinicians’ favorable attitudes toward the intervention. This reflects overall positive attitudes and an eagerness to incorporate technology into practice found in research on clinicians’ attitudes toward technology in mental health care [78]. This is important considering that there is research highlighting that clinicians’ attitudes are pivotal in intervention implementation [79].

A barrier to the implementation of blended interventions that required clinician involvement was the lack of engagement that people who self-harm have with mental health services. This is consistent with the body of literature that corroborates the lack of professional help-seeking among people who self-harm [8-11] and further emphasizes the need for mHealth interventions for self-harm that individuals can access easily and discretely. Despite many studies having positive outcomes, it seems that not many of the interventions studied are readily available to the general public or even to those attending mental health services. Moreover, few papers made this information apparent, so information on availability was sought from internet and app store searches and by contacting the authors of the papers.

### Implications for Future Research

This review identified several limitations in the current literature on mHealth tools for managing self-harm. Notably, research thus far has been limited to White adult females from western societies, yet self-harm has been identified as a significant issue among minority groups [68,69], highlighting the need to diversify research by recruiting understudied groups such as males and minority populations who may also benefit from mHealth interventions. It is also important for future studies to report the ethnicity of participants. Similarly, participants have mostly been from clinical samples; however, given that people who self-harm do not often seek professional help, it is possible that findings from clinical samples may not necessarily be generalizable to wider populations who self-harm. Therefore, it is important to assess the efficacy of interventions in community samples. It is also essential for more intervention developers to collaborate with people who have lived experience of self-harm, given the limited instances thus far highlighted in this review.

Another notable point is that the measurements of self-harm recovery were typically restricted to a reduction in the frequency of episodes and, although this may be a useful assessment, it may also be worth considering measuring other elements as well, such as the severity of self-harming episodes or any other substitute self-destructive behaviors.

Another consideration to take forward from this review is the reliance on RCTs to evaluate digital interventions. Although RCTs have long been considered the gold standard method, the pace at which digital interventions develop and evolve means that the data can be outdated before the trial has been completed [80-82]. Following this, it may be prudent for future research to also consider different study designs, such as pre- and posttests that can keep up with the rapid development of digital interventions. Furthermore, RCTs can indicate whether an intervention is effective but may be limited in their ability to explore the reason [83]. Therefore, it may also be beneficial for future research to apply the NIMH’s experimental therapeutics approach or use qualitative studies that can contribute further to the understanding of how and why certain interventions are effective as well as whether they are safe and do no harm. Similarly, the application of the experimental therapeutic approach may contribute further to the understanding of how and why certain interventions are effective [84]. Future work should also focus on the implementation and dissemination of effective interventions, following the lack of availability of interventions within the papers in this review.

### Limitations

As the purpose of this review was to collate the data available on mHealth tools for managing self-harm, there was no scope to conduct a quality assessment of the included studies. This means that we cannot verify that the research included here is of sufficient quality to draw concrete inferences from. This review may also have been limited by the decision to not include any papers for which there was no English version available. Nonetheless, this review offers insight into the current evidence base for mHealth interventions for self-harm.

### Conclusions

This review has synthesized the current evidence for mHealth tools for managing self-harm. Overall, there are useful interventions that have been developed to promote recovery from self-harm. However, certain limitations pose challenges in drawing firm conclusions from the included studies. Suggestions for how future research can improve upon this have been made, in the hope of developing a robust evidence base so that clinicians and users are better equipped to make informed decisions about which mHealth tools to use. This will hopefully help to overcome some of the barriers that people who self-harm face in accessing support.

## Appendix

Multimedia Appendix 1 Full search strategy.

## Abbreviations

mHealth: mobile health
RCT: randomized controlled trial
