# Supplementary material for: mHealth Interventions for Self-Harm: Scoping Review
Source: J Med Internet Res. 2021 Apr 30;23(4):e25140. doi: 10.2196/25140 (PMC8122298; doi:10.2196/25140)
Supplement: Multimedia Appendix 1 [file jmir_v23i4e25140_app1.doc]

## Appendix 1

### Search terms

self?injur* OR "self?harm*" OR "self?poison*" OR "self?cut*" OR "self?mutilat*" OR “auto?mutilat*” OR “NSSI” OR "non?suicidal self?injur*" OR “DSH” OR “deliberate self?harm*” OR “intentional self?harm*”AND “mHealth” OR "digital intervention*" OR "smartphone app*" OR “mobile app*” OR “mobile health” OR “mobile tech*” OR “Android” OR “Apple” OR “iPhone” OR “iOS” OR “Samsung” OR “iPad” OR “tablet” OR “cell phone” OR “personal digital assistant” OR “PDA” OR “smartphone” OR “mobile phone” OR “SMS” OR “text messag*” OR “windows phone” OR “mobile device” OR “smart?watch” OR “apple watch” OR “fitbit”

### Database search formats

Embase: ('self?injur*':ti,ab,kw OR 'self?harm*':ti,ab,kw OR 'self?poison*':ti,ab,kw OR 'self?cut*':ti,ab,kw OR 'self?mutilat*':ti,ab,kw OR 'auto?mutilat*':ti,ab,kw OR 'nssi':ti,ab,kw OR 'non?suicidal self?injur*':ti,ab,kw OR 'dsh':ti,ab,kw OR 'deliberate self?harm*':ti,ab,kw OR 'intentional self?harm*':ti,ab,kw) AND ('mhealth':ti,ab,kw OR 'digital intervention*':ti,ab,kw OR 'smartphone app*':ti,ab,kw OR 'mobile app*':ti,ab,kw OR 'mobile health':ti,ab,kw OR 'mobile tech*':ti,ab,kw OR 'android':ti,ab,kw OR 'apple':ti,ab,kw OR 'iphone':ti,ab,kw OR 'ios':ti,ab,kw OR 'samsung':ti,ab,kw OR 'ipad':ti,ab,kw OR 'tablet':ti,ab,kw OR 'cell phone':ti,ab,kw OR 'personal digital assistant':ti,ab,kw OR 'pda':ti,ab,kw OR 'smartphone':ti,ab,kw OR 'mobile phone':ti,ab,kw OR 'sms':ti,ab,kw OR 'text messag*':ti,ab,kw OR 'windows phone':ti,ab,kw OR 'mobile device':ti,ab,kw OR 'smart?watch':ti,ab,kw OR 'apple?watch':ti,ab,kw OR 'fitbit':ti,ab,kw)

PubMed: (("self?injur*" OR "self?harm*" OR "self?poison*" OR "self?cut*" OR "self?mutilat*" OR “auto?mutilat*” OR “NSSI” OR "non?suicidal self?injur*" OR “DSH” OR “deliberate self?harm*” OR “intentional self?harm*”)) AND ("mHealth” OR "digital intervention*" OR "smartphone app*" OR “mobile app*” OR “mobile health” OR “mobile tech*” OR “Android” OR “Apple” OR “iPhone” OR “iOS” OR “Samsung” OR “iPad” OR “tablet” OR “cell phone” OR “personal digital assistant” OR “PDA” OR “smartphone” OR “mobile phone” OR “SMS” OR “text messag*” OR “windows phone” OR “mobile device” OR “smart?watch” OR “apple?watch” OR “fitbit”)

PsycInfo: Any Field: "self?injur*" OR "self?harm*" OR "self?poison*" OR "self?cut*" OR "self?mutilat*" OR “auto?mutilat*” OR “NSSI” OR "non?suicidal self?injur*" OR “DSH” OR “deliberate self?harm*” OR “intentional self?harm*” AND Any Field: "mHealth” OR "digital intervention*" OR "smartphone app*" OR “mobile app*” OR “mobile health” OR “mobile tech*” OR “Android” OR “Apple” OR “iPhone” OR “iOS” OR “Samsung” OR “iPad” OR “tablet” OR “cell phone” OR “personal digital assistant” OR “PDA” OR “smartphone” OR “mobile phone” OR “SMS” OR “text messag*” OR “windows phone” OR “mobile device” OR “smart?watch” OR “apple?watch” OR “fitbit”

PsycExtra: Any Field: "self?injur*" OR "self?harm*" OR "self?poison*" OR "self?cut*" OR "self?mutilat*" OR “auto?mutilat*” OR “NSSI” OR "non?suicidal self?injur*" OR “DSH” OR “deliberate self?harm*” OR “intentional self?harm*” AND Any Field: "mHealth” OR "digital intervention*" OR "smartphone app*" OR “mobile app*” OR “mobile health” OR “mobile tech*” OR “Android” OR “Apple” OR “iPhone” OR “iOS” OR “Samsung” OR “iPad” OR “tablet” OR “cell phone” OR “personal digital assistant” OR “PDA” OR “smartphone” OR “mobile phone” OR “SMS” OR “text messag*” OR “windows phone” OR “mobile device” OR “smart?watch” OR “apple?watch” OR “fitbit”

PsycArticles: Any Field: "self?injur*" OR "self?harm*" OR "self?poison*" OR "self?cut*" OR "self?mutilat*" OR “auto?mutilat*” OR “NSSI” OR "non?suicidal self?injur*" OR “DSH” OR “deliberate self?harm*” OR “intentional self?harm*” AND Any Field: "mHealth” OR "digital intervention*" OR "smartphone app*" OR “mobile app*” OR “mobile health” OR “mobile tech*” OR “Android” OR “Apple” OR “iPhone” OR “iOS” OR “Samsung” OR “iPad” OR “tablet” OR “cell phone” OR “personal digital assistant” OR “PDA” OR “smartphone” OR “mobile phone” OR “SMS” OR “text messag*” OR “windows phone” OR “mobile device” OR “smart?watch” OR “apple?watch” OR “fitbit”

Web of Science: TOPIC: ("self?injur*" OR "self?harm*" OR "self?poison*" OR "self?cut*" OR "self?mutilat*" OR “auto?mutilat*” OR “NSSI” OR "non?suicidal self?injur*" OR “DSH” OR “deliberate self?harm*” OR “intentional self?harm*”) AND TOPIC: ("mHealth” OR "digital intervention*" OR "smartphone app*" OR “mobile app*” OR “mobile health” OR “mobile tech*” OR “Android” OR “Apple” OR “iPhone” OR “iOS” OR “Samsung” OR “iPad” OR “tablet” OR “cell phone” OR “personal digital assistant” OR “PDA” OR “smartphone” OR “mobile phone” OR “SMS” OR “text messag*” OR “windows phone” OR “mobile device” OR “smart?watch” OR “apple?watch” OR “fitbit”)

Cochrane Library: self?injur* OR "self?harm*" OR "self?poison*" OR "self?cut*" OR "self?mutilat*" OR “auto?mutilat*” OR “NSSI” OR "non?suicidal self?injur*" OR “DSH” OR “deliberate self?harm*” OR “intentional self?harm*” in Title Abstract Keyword AND mHealth OR "digital intervention*" OR "smartphone app*" OR “mobile app*” OR “mobile health” OR “mobile tech*” OR Android OR Apple OR iPhone OR iOS OR Samsung OR iPad OR tablet OR “cell phone” OR “personal digital assistant” OR PDA OR smartphone OR “mobile phone” OR SMS OR “text messag*” OR “windows phone” OR “mobile device” OR “smart?watch” OR “apple?watch” OR fitbit in Title Abstract Keyword - (Word variations have been searched).
